# Advances in mechanisms and challenges in clinical translation of synergistic nanomaterial-based therapies for melanoma

**DOI:** 10.3389/fcell.2025.1648379

**Published:** 2025-07-25

**Authors:** Yibo Zhang, Xilin Liu, Guangzhi Wu

**Affiliations:** ^1^Department of Wound Repair, Plastic and Reconstructive Microsurgery, China-Japan Union Hospital of Jilin University, Changchun, China; ^2^Department of Hand and Foot surgery, China-Japan Union Hospital of Jilin University, Changchun, China

**Keywords:** melanoma, nanomaterials, synergistic therapy, precision medicine, clinical translation

## Abstract

Melanoma is a highly malignant form of skin cancer, with its incidence and mortality rates continuously rising on a global scale. Although traditional treatments such as surgery, chemotherapy, radiotherapy, as well as targeted and immunotherapy, have made certain progress, the efficacy of these therapeutic modalities remains limited due to the high metastatic potential, heterogeneity, and drug resistance of melanoma. In recent years, nanomaterials, with their unique physicochemical properties, have emerged as a significant research focus in tumor therapy. Nanomaterials can enhance the targeted delivery of drugs, increase drug accumulation in tumors, and reduce side effects, and they have shown great potential in the synergistic treatment of melanoma. This review summarizes the mechanistic breakthroughs of nanomaterials in the synergistic treatment of melanoma, including the combined application of nanocarriers in photothermal therapy, photodynamic therapy, and immunotherapy. It also explores how precise drug delivery can improve therapeutic efficacy and overcome tumor immune evasion and drug resistance. Furthermore, the challenges faced in the clinical translation of nanomaterial-based synergistic treatment are discussed, such as biosafety, delivery efficiency, and the need for personalized treatment. Despite these challenges, the continuous development of nanotechnology offers new hope for the comprehensive treatment of melanoma and lays the foundation for the realization of precision medicine in the future.

## 1 Introduction

Melanoma is a highly malignant form of skin cancer, with its incidence and mortality rates continuously increasing worldwide (Beberok et al., 2020; Möller et al., 2020; Lopes et al., 2022b; Zhu et al., 2022). In 2022, there were approximately 330,000 newly diagnosed cases of melanoma globally, resulting in around 58,000 deaths. The incidence is predominantly concentrated in regions with predominantly Caucasian populations, such as Australia, North America, and Europe, while it remains relatively lower in Asia and Africa (Wang et al., 2025). It is projected that over the next 20 years, the global incidence and mortality rates of melanoma will increase by 70%–80%, respectively (Lopes et al., 2022b). Owing to the highly metastatic and heterogeneous nature of melanoma, conventional therapeutic modalities, such as surgery, chemotherapy, and radiotherapy, have demonstrated limited efficacy in controlling advanced disease, and are often associated with severe toxic side effects and drug resistance issues (Czarnecka A. M. et al., 2020; Jaune et al., 2021; Chen et al., 2022; Cunha et al., 2022). Although targeted therapy and immune checkpoint inhibitors have significantly improved the survival of patients with advanced melanoma in recent years, the majority of patients ultimately face drug tolerance and severe adverse reactions, resulting in suboptimal long-term therapeutic outcomes (Cunha et al., 2022; Lopes et al., 2022b; Zheng et al., 2022). Therefore, there is an urgent need to explore novel therapeutic strategies, such as nanomaterial-based synergistic treatments, to address these challenges (Lopes et al., 2022b; Sekar et al., 2023).

Nanomaterials are generally defined as materials with at least one dimension in the nanoscale range (approximately 1–100 nm), and their unique size and high surface area-to-volume ratio endow them with significantly different physical, chemical, and biological properties compared to bulk materials (Cheng et al., 2022; Allami et al., 2023; Sell et al., 2023). These characteristics confer nanomaterials with distinct advantages in tumor therapy, including enhanced tumor permeability and drug accumulation effects (such as the enhanced permeability and retention (EPR) effect), high drug-loading capacity, and controllable targeting delivery properties (Giri et al., 2023; Kida et al., 2023; Sell et al., 2023). In the treatment of melanoma, nanomaterials can effectively deliver therapeutic agents precisely to the lesion site, reducing systemic toxicity and significantly improving therapeutic efficacy (Mazloum-Ravasan et al., 2022; Shah et al., 2022; Yu et al., 2023). Most importantly, nanomaterials can integrate multiple therapeutic modalities simultaneously (Kostiv et al., 2020; Chen et al., 2022; Gollavelli et al., 2022), such as the synergistic strategy combining photothermal therapy with immunotherapy, which has demonstrated a significant advantage in effectively inhibiting tumor growth and metastasis in animal experiments (Xue et al., 2022; Lu et al., 2023).

Chemotherapeutic-photothermal synergistic therapy based on nanocarriers has also shown synergistically enhanced antitumor efficacy, far exceeding the therapeutic outcomes of monotherapy (Lu et al., 2023; Peltek et al., 2023). This dual-modality approach addresses some of the most important drawbacks of traditional cancer treatments by utilizing the special qualities of nanocarriers to enhance tumor targeting, decrease systemic toxicity, and improve drug delivery (Zheng et al., 2020; Tang L. et al., 2021). Combining photothermal therapy (PTT) and chemotherapy on a single nanoplatform enables precise control over drug release and localized heat generation, which can enhance the therapeutic effect in concert (Li et al., 2018; Bi et al., 2024). When exposed to near-infrared (NIR) radiation, gold-based nanoparticles, like Her2-DOX-SPIOs@PLGA@Au NPs, can produce heat and deliver chemotherapeutic agents like doxorubicin (DOX), which can cause immunogenic cell death (ICD) and tumor microenvironment remodeling (Chen et al., 2023). This approach not only enhances the accessibility of therapeutic agents to tumor cells but also activates immune responses, further contributing to tumor eradication (Shan et al., 2021).

Stimulus-responsive components, like pH-sensitive or photothermal-responsive materials, are frequently incorporated into the creative design of these nanocarriers to allow for controlled drug release in the tumor microenvironment. Camptothecin (CPT) nanorods coated with polydopamine (PDA) (CNR@PDA-CM) have shown improved tumor accumulation and circulation stability, attaining a nearly 5-fold increase in tumor accumulation and a 73-fold longer terminal half-life in comparison to free CPT (Wang Q. et al., 2021). In metastatic breast cancer models, PDA’s photothermal effect not only speeds up CPT release but also enhances drug permeability inside the tumor, producing strong and complementary therapeutic results (Liu et al., 2024). Mitoxantrone (MTO)-based multifunctional nanomicelles have been created to combine mild PTT with chemotherapy. The heat produced increases DNA damage and inhibits heat shock protein 70 (HSP70), which lowers tumor thermoresistance and increases treatment effectiveness (Zhu et al., 2019).

The ability of chemotherapeutic-photothermal synergistic therapy to overcome drug resistance, a significant obstacle in the treatment of cancer, is one of its main benefits. It has been demonstrated that PTT and low-dose chemotherapy increase the immunogenicity of tumor cells, resulting in elevated expression of calreticulin (CRT) and heat shock proteins (HSP70), which facilitate immune recognition and activation (Bruno et al., 2017). A multifaceted strategy for tumor eradication is created by this immunomodulatory effect in addition to the direct cytotoxic effects of chemotherapy and hyperthermia. Significant tumor growth inhibition and immune activation have been shown when Her2-targeted nanoparticles are used in Her2-positive breast cancer models, underscoring the strategy’s potential to overcome resistance mechanisms (Steinbrueck et al., 2020). These nanocarriers’ incorporation of imaging technologies, such as photoacoustic (PA) and magnetic resonance imaging (MRI), enables accurate localization of therapeutic agents and real-time tumor response monitoring. In addition to improving treatment accuracy, this theranostic approach offers important insights into the dynamics of immune activation and tumor microenvironment remodeling (Zhang et al., 2024). Successful validation of Her2-DOX-SPIOs@PLGA@Au NPs for PA/MRI dual-modal imaging shows their potential as both therapeutic and diagnostic agents (Kato et al., 2024).

Innovation in this field is further demonstrated by the creation of carrier-free co-delivery systems, such as DiR nanoassemblies and gambogic acid (GA). By doing away with the need for conventional carriers, these systems lower the risk of toxicity and increase the effectiveness of drug loading. It has been demonstrated that the precise co-administration of the heat shock protein 90 (HSP90) inhibitor GA and the photothermal photosensitizer DiR increases the effectiveness of PTT by making tumor cells more sensitive to hyperthermia and causing a notable tumor regression in preclinical models (Phour et al., 2022). Self-delivery systems for chemotherapeutic medications and photosensitizers, like podophyllotoxin (PPT) and pyropheophorbide a (PPa), which have synergistic antitumor effects and enhanced tumor accumulation, have been developed using computer-aided design techniques (Courneya et al., 2025). A very promising approach to treating cancer is chemotherapeutic-photothermal synergistic therapy based on nanocarriers, which offers improved antitumor efficacy, decreased systemic toxicity, and the capacity to overcome drug resistance. The development of carrier-free and stimuli-responsive systems further improves the accuracy and efficacy of this therapy, while the combination of chemotherapy, PTT, and imaging capabilities within a single nanoplatform offers a comprehensive approach to tumor eradication. In order to improve outcomes for cancer patients, future research should concentrate on refining the design of these nanocarriers, investigating their potential in conjunction with other therapeutic modalities, and advancing their clinical translation (Smith et al., 2024; Hoeppner et al., 2025). This review will provide a detailed account of the current bottlenecks in melanoma treatment and how nanomaterials can achieve synergistic therapeutic effects at the molecular and cellular levels through combined treatment strategies, resulting in a novel therapeutic outcome.

## 2 Limitations of traditional therapeutic modalities

### 2.1 Chemotherapy and radiotherapy

Melanoma exhibits poor responsiveness to conventional chemotherapy and radiotherapy, with limited therapeutic efficacy (Tang K. et al., 2021; Natarelli et al., 2024). The response rate to monotherapy with classic chemotherapeutic agents, such as dacarbazine, is below 10%, and it fails to significantly prolong patient survival (Mundra et al., 2015). Chemotherapeutic drugs lack tumor selectivity, and their nonspecific cytotoxicity to normal tissues results in severe adverse effects, including fatigue, nausea, and alopecia (Cunha et al., 2022; Natarelli et al., 2024). Given the suboptimal efficacy and significant toxic side effects, the role of chemotherapy and radiotherapy in melanoma treatment has gradually been supplanted by targeted therapy and immunotherapy, being reserved as adjunctive therapies in advanced disease or when other modalities fail (Boer et al., 2019; Takahashi and Nagasawa, 2020; Natarelli et al., 2024). Radiotherapy can be used for local control; however, it is ineffective in achieving a cure for advanced metastatic melanoma (Takahashi and Nagasawa, 2020; Natarelli et al., 2024).

The complicated biology of melanoma and the difficulties in attaining successful therapeutic results are reflected in the various limitations of chemotherapy and radiation therapy in the treatment of the disease. Because melanoma is notoriously resistant to traditional treatments, especially when it is advanced, alternative therapeutic approaches have had to be investigated (Aninditha et al., 2019). Because of the tumor’s inherent resistance mechanisms and the high mutational burden that propels its progression, chemotherapy, which has historically been used to treat a variety of cancers, has demonstrated limited efficacy in melanoma (Wu et al., 2023). Although chemotherapy drugs like dacarbazine have been used, their usefulness in clinical practice is limited by their low response rates and frequent severe toxicity (Hendrickx et al., 2020). Despite its effectiveness in managing localized disease, radiotherapy encounters difficulties in treating melanoma because of the tumor’s relative radioresistance. Because melanoma cells have a high capacity for DNA repair, photon-based radiation therapy is less effective (Cho et al., 2018).

The requirement for high doses to control tumors adds to radiotherapy’s limitations by raising the possibility of harming nearby healthy tissues (Khan et al., 2018). In cases of brain metastases, where the sensitive neural tissue is extremely vulnerable to radiation-induced toxicity, this is especially problematic (Verduin et al., 2017). The availability of specialized facilities and the high cost of treatment limit the widespread use of heavy ion therapy, which has demonstrated promise in overcoming radioresistance due to its higher relative biological effectiveness (RBE) with carbon (C12) and oxygen (O16) ions (Smart et al., 2024). To improve the effectiveness of treatment, the combination of radiation and systemic therapies, like immunotherapy, has also been investigated. The order and timing of these treatments are still up for debate, though, as some research indicates that immune checkpoint inhibitors (ICIs) and previous radiation therapy may not substantially enhance results (Teterycz et al., 2020). The incapacity of chemotherapy to address the heterogeneity of melanoma, which frequently results in the development of drug resistance, is another example of its limitations. This resistance is greatly influenced by the tumor microenvironment (TME), which can encourage tumor survival and immune evasion (Saiag et al., 2022). The integration of these therapies is made more difficult by the possibility of immune escape due to the upregulation of programmed death-ligand 1 (PD-L1) in melanoma cells after radiation therapy (Knispel et al., 2020). Myelosuppression and gastrointestinal side effects are among the systemic toxicity of chemotherapy that frequently require dose reductions or treatment discontinuation, which can jeopardize therapeutic results (Long et al., 2023).

When it comes to mucosal melanoma (MM), a rare and aggressive subtype of melanoma that develops in mucosal membranes, the limitations of both chemotherapy and radiation therapy are especially noticeable. Compared to cutaneous melanoma, MM exhibits lower mutation rates, which could explain why it responds less well to systemic treatments (Department of Error, 2023). The overall effectiveness of radiotherapy is limited by the high rates of distant metastasis and the absence of standardized treatment protocols, even though it has been used to achieve local control in MS (Welch et al., 2021). There have been conflicting findings regarding the combination of radiotherapy and immunotherapy in MM; some studies have found no discernible benefit, while others have reported improved progression-free survival (PFS) when radiotherapy is coupled with anti-PD-1 therapy (Grover et al., 2025).

The intrinsic resistance mechanisms of the tumor, the difficulties in achieving efficient local and systemic control, and the high toxicity of these treatments are the main reasons why chemotherapy and radiation therapy are not always effective in treating melanoma. Although new treatment options have been made possible by developments in immunotherapy and targeted therapies, integrating these modalities with traditional therapies is still difficult and needs more research. Future studies should concentrate on finding biomarkers to forecast how well a treatment will work, streamlining the sequencing and combination of treatments, and creating innovative approaches to get past melanoma resistance mechanisms.

### 2.2 Targeted therapy

Targeted therapies directed against the BRAF/MEK pathway can achieve significant initial responses in patients harboring specific mutations, but the therapeutic effects are often not durable (Mundra et al., 2015). The majority of patients develop resistance within 6–7 months, leading to disease progression (Mundra et al., 2015). Additionally, the tolerability and cutaneous toxicities associated with targeted agents limit their long-term monotherapy use (Mundra et al., 2015). Therefore, even with the emergence of new targeted agents against additional mutation targets, monotherapy with targeted drugs typically needs to be combined with other therapeutic modalities to delay the onset of resistance (Natarelli et al., 2024).

### 2.3 Immunotherapy

Immunotherapy, particularly immune checkpoint inhibitors (such as anti-PD-1 and anti-CTLA-4 antibodies), has brought revolutionary advancements in the treatment of advanced melanoma, achieving durable remission in a considerable proportion of patients. However, approximately 40%–60% of patients remain unresponsive to immunotherapy or develop secondary resistance (Natarelli et al., 2024). Immunotherapy significantly enhances the immune system’s ability to attack tumor cells, thereby exerting substantial selective pressure. This can lead to tumor resistance or immune evasion, as tumors may downregulate antigen expression or recruit immunosuppressive cells to escape immune clearance (Asher et al., 2019; Huang and Zappasodi, 2022; Lim et al., 2023). Consequently, some patients experience disease recurrence after initial response, or exhibit primary non-responsiveness to immunotherapy, posing a significant clinical challenge (Lim et al., 2023). Additionally, immunotherapy itself may induce immune-related adverse events (such as autoimmune inflammation), necessitating a careful balance between therapeutic efficacy and safety (Asher et al., 2019; Izawa et al., 2022).

## 3 Biological characteristics of melanoma

### 3.1 High metastatic potential and proneness to recurrence

Melanoma is a highly aggressive cutaneous malignancy with a strong propensity for metastasis, and patients with advanced disease have a particularly poor prognosis (Marie et al., 2020; Natarelli et al., 2024). The 5-year survival rate for patients with metastatic melanoma is less than 15% (Mundra et al., 2015). Surgical resection is only effective for early-stage, localized lesions; however, once the disease progresses to stage III/IV and metastasis occurs, conventional therapeutic approaches often fail to completely eradicate the tumor foci, thereby increasing the likelihood of recurrence (Natarelli et al., 2024). Melanoma cells are capable of surviving under therapeutic pressure and entering a dormant state, which can lead to the reinitiation of proliferation and subsequent tumor relapse. Resistance to traditional chemotherapy further complicates the management of recurrent disease (Špaková et al., 2021; Natarelli et al., 2024). Studies have demonstrated that chemoresistance can drive disease progression and recurrence (Palušová et al., 2020; Natarelli et al., 2024), which is a significant cause of repeated treatment failures in melanoma therapy.

A complex interaction of genetic, molecular, and microenvironmental factors affects how melanoma spreads. About 20% of malignant melanomas spread, frequently by lymphatic or hematogenous pathways, and extramammary metastases, especially from melanoma (29.8%), frequently occur in the breast (El-Tani et al., 2016). Histopathological examination and immunohistochemistry are necessary for a conclusive diagnosis of metastatic melanoma because of its varied radiographic presentations, which can resemble benign lesions or primary mammary adenocarcinoma (El-Tani et al., 2016). While no single immunohistochemical marker is completely specific or sensitive, S-100, Melan-A, HMB-45, and tyrosinase are essential for differentiating melanoma from other cancers (El-Tani et al., 2016).

Melanoma’s molecular landscape is marked by considerable heterogeneity, including loss of tumor suppressors like PTEN and CDKN2A, mutations in important oncogenes like BRAF, NRAS, and KIT, and other changes that promote tumor growth and metastasis (Kreuger et al., 2023). ARHGAP35 is a novel driver gene linked to immune suppression and metastasis in melanoma, according to recent research. Mutations in ARHGAP35 are associated with a poor prognosis and decreased adaptive immune responses, especially in naïve CD4^+^ T cells and myeloid dendritic cells, which are essential for antitumor immunity (Li et al., 2022). This discovery emphasizes how genetic changes influence the tumor microenvironment and promote the spread of metastases. Treatment plans are further complicated by the fact that MDM2 and MDM4 amplifications, which dysregulate the TP53 pathway, have been linked to increased rates of liver and brain metastases (Kreuger et al., 2023).

Melanoma’s propensity to recur even after surgical resection is another indication of its high potential for metastasis. A subset of patients with thin melanomas (Breslow thickness ≤1 mm) have a significantly higher risk of metastasis and recurrence, underscoring the need for accurate prognostic markers (Richetta et al., 2018). Elevated mitotic rate (MR) in non-ulcerated thin melanomas is associated with higher sentinel lymph node positivity and recurrence rates, making it a significant prognostic factor (Ly et al., 2023). To identify patients at a higher risk of disease progression, these findings imply that MR should be taken into account when staging and managing early-stage melanoma (Ly et al., 2023).

Key signaling pathways that support cell proliferation, survival, and angiogenesis, like MAPK and PI3K/AKT, are frequently activated in the development of metastatic melanoma (Kreuger et al., 2023). In patients with BRAF-mutant melanoma, targeted therapies such as BRAF and MEK inhibitors have demonstrated effectiveness in blocking these pathways and enhancing progression-free survival (Kudchadkar et al., 2020). The investigation of combination therapies and immune-modulating agents is necessary, though, because resistance to targeted therapies continues to be a major obstacle. Anti-PD-1 and anti-CTLA-4 antibodies are examples of immune checkpoint inhibitors that have transformed the treatment of metastatic melanoma by boosting antitumor immune responses. In patients with advanced melanoma, ipilimumab has been shown to provide a 20% reduction in recurrence risk and a sustained overall survival (El-Tani et al., 2016). Patients with low metastasis scores (MET scores) have demonstrated promise with anti-PD-1 therapy, indicating that it may be used as an adjuvant treatment to prevent metastasis (Shen et al., 2023).

The development and recurrence of melanoma are significantly influenced by the TME. An immunosuppressive TME with increased regulatory T cell infiltration and decreased CD8^+^ T cell activity is a hallmark of metastatic melanoma and is associated with a worse prognosis (Shen et al., 2023). TME-modulating techniques, like immune vaccines that target melanoma-specific antigens (like MART1/Melan-A, gp100, and tyrosinase), have demonstrated promise in boosting cytotoxic T cell responses and enhancing survival in patients who respond (El-Tani et al., 2016). A complex interaction between genetic mutations, signaling pathway activation, and immune microenvironment modulation drives melanoma’s high metastatic potential and propensity for recurrence. There are still issues like treatment resistance and the requirement for accurate prognostic indicators. To enhance the prognosis of patients with metastatic and recurrent melanoma, future studies should concentrate on finding new biomarkers, creating combination treatments, and refining immune-modulating techniques.

### 3.2 Immunosuppressive tumor microenvironment

A complex ecosystem, the immunosuppressive TME in melanoma is defined by the interaction of different immune cell populations, cytokines, and immune checkpoint molecules, all of which work together to promote tumor growth and immunotherapy resistance (Anagnostou et al., 2020; Zhang et al., 2022). Immune checkpoint molecules like PD-1, CTLA-4, LAG-3, and TIM-3 are essential to this immunosuppressive environment. They are overexpressed in melanoma and prevent T cell activation, which makes immune evasion easier (Schmitt et al., 2021; Bogéa et al., 2022). Although their blockade has demonstrated clinical efficacy in treating melanoma, the suppression of T cell-mediated anti-tumor responses is largely dependent on PD-1 and its ligand PD-L1, which have limited response rates because of intrinsic and acquired resistance mechanisms (Kakizaki et al., 2015; Jorge et al., 2020). Targeting LAG-3 and TIM-3 to increase the effectiveness of immune checkpoint blockade is being investigated. These co-inhibitory receptors have been shown to work in concert with PD-1 to suppress T cell function (Chen et al., 2017; Deng et al., 2021). By encouraging the differentiation of regulatory T cells (Tregs) and preventing the activity of cytotoxic T lymphocytes (CTLs) and dendritic cells (DCs), cytokines like IL-10, TGF-β, and IDO worsen immunosuppression (Georgoulias and Zaravinos, 2022; Hossain et al., 2022) By reducing tryptophan and generating immunosuppressive metabolites, IDO, in particular, is upregulated in melanoma and promotes immune tolerance (Zhou et al., 2019).

The immunosuppressive landscape is significantly shaped by the makeup of immune cell populations within the TME. Melanoma is enriched in tumor-associated macrophages (TAMs), especially the M2 subtype, which stimulate tumor growth by preventing T cell infiltration and releasing immunosuppressive cytokines (Falcomatà et al., 2023; Xing et al., 2024). The presence of regulatory T cells (Tregs), another important element of the immunosuppressive TME, is associated with a poor prognosis and resistance to immune checkpoint inhibitors (Kudchadkar et al., 2020; Zhang et al., 2025). The infiltration of natural killer (NK) cells and cytotoxic CD8^+^ T cells, on the other hand, is linked to better immunotherapy outcomes, underscoring the significance of cultivating an immune-active TME (Pang et al., 2023; Jin et al., 2024). Exhausted T cells express high levels of immune checkpoint molecules and have decreased cytotoxic activity, which makes the therapeutic landscape more complex due to the heterogeneity of CD8^+^ T cell subpopulations, including exhausted and terminally differentiated states (Qin et al., 2023; Yang et al., 2025). According to recent research, intratumoral B cell infiltration may improve responses to immune checkpoint blockade, highlighting the role of B cells and tertiary lymphoid structures in fostering anti-tumor immunity (Shen et al., 2024).

Tumor-intrinsic characteristics like mutations in JAK1/JAK2, PTEN loss, and WNT/β-catenin signaling, which alter antigen presentation and T cell recruitment, further impact the dynamic interaction between tumor cells and the immune system (Bellucci et al., 2013; Hu et al., 2021). Mutations in B2M and major histocompatibility complex (MHC) class I are examples of defects in the antigen presentation machinery that allow tumor cells to avoid T cell recognition and increase resistance to immunotherapy (Torrejon et al., 2023; Han et al., 2025). Furthermore, the neoantigen load and tumor mutation burden (TMB) are important factors that determine the response to immunotherapy because higher TMB is linked to better clinical results and increased neoantigen generation (Chan et al., 2019; Szeto et al., 2020). The anti-tumor immune response can be weakened by immunoediting mechanisms like neoantigen loss and downregulation of interferon-ϣ signaling, which emphasizes the necessity of integrated models to forecast treatment results (Jorgovanovic et al., 2020; Galassi and Galluzzi, 2023).

Targeting new immune checkpoints, adjusting cytokine networks, and reprogramming the TME to improve immune cell infiltration and function are some of the emerging methods for overcoming immunosuppression in melanoma. Although they are linked to higher toxicity, combination therapies that block PD-1 and CTLA-4 at the same time have demonstrated improved efficacy when compared to monotherapy (Willsmore et al., 2021). Small-molecule inhibitors that target TGF-β or IDO signaling are also being investigated as a way to reverse immunosuppression and increase the effectiveness of immune checkpoint blockade (Guo et al., 2021; Leonardo-Sousa et al., 2025). By boosting T cell-mediated immunity, nanotechnology-based strategies, like lysosomal-targeting nanoparticles, have also shown promise in causing immunogenic cell death and turning “cold” tumors into “hot” ones (Giustarini et al., 2021; Wu et al., 2024). In order to determine patient-specific therapeutic targets and forecast immunotherapy responses, personalized proteogenomic approaches that combine genomic, transcriptomic, and proteomic data are being developed (Huber et al., 2024).

Immune checkpoint molecules, cytokines, and immune cell populations interact to create the immunosuppressive TME in melanoma, which is a complex obstacle to successful immunotherapy. To improve treatment outcomes and overcome resistance to immune checkpoint blockade, it is essential to comprehend the intricate mechanisms underlying immunosuppression and create novel approaches to modulate the TME. To fully utilize the immune system in the fight against melanoma, future studies should concentrate on combining multi-omics data, investigating new therapeutic targets, and refining combination therapies.

### 3.3 Lack of targeted and penetrating drug delivery

**FIGURE 1:**
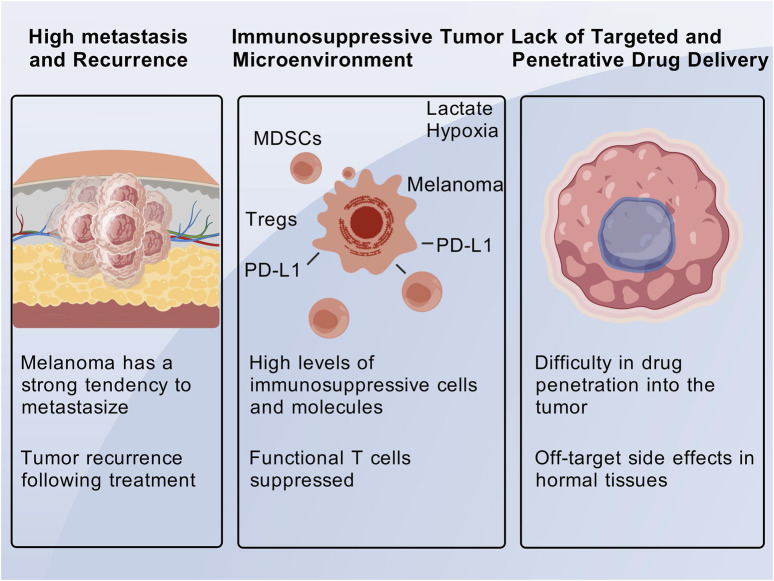
The biological characteristics of tumors bring about significant challenges.

Melanoma is often characterized by high intratumoral heterogeneity and dense tumor stroma, which collectively impede the efficient penetration and uniform distribution of conventional drugs within the tumor mass (Mundra et al., 2015; Mejbel et al., 2019). The abnormal structure of tumor vasculature, coupled with areas of poor perfusion, further restricts the delivery of systemically administered drugs to all tumor cells (Mundra et al., 2015; Mikoshiba et al., 2019). Insufficient drug concentration at the tumor site and excessive exposure in normal tissues not only reduce therapeutic efficacy but also exacerbate toxicity. The lack of tumor specificity is a major limitation of traditional drugs: for instance, chemotherapeutic agents distribute systemically and nonspecifically kill proliferating cells, leading to adverse effects such as myelosuppression and mucositis (Jin et al., 2019; Natarelli et al., 2024). Even targeted therapies may engage homologous pathways in normal cells, resulting in off-target side effects. Regarding drug resistance, melanoma cells can develop resistance to monotherapy through genetic mutations or pathway reprogramming (Amaral et al., 2020), such as the activation of bypass signaling pathways that lead to resistance against BRAF inhibitors (Molnár et al., 2019). This highlights the necessity for developing strategies that can simultaneously target multiple pathways to overcome resistance (Figure 1).

## 4 New directions for nanomaterial-based tumor therapy research

In light of the aforementioned limitations, there is a clinical imperative for more effective and selective therapeutic modalities that can eradicate primary lesions while preventing metastasis and recurrence. There is a need for technologies that can precisely deliver drugs to tumors while minimizing damage to normal tissues, thereby enhancing tumor cell killing while reducing side effects (Mundra et al., 2015). Increasing the accumulation and penetration depth of drugs within melanoma tissue, while avoiding clearance by normal organs, represents a crucial research direction. Given the propensity of melanoma for mutation and drug resistance, combination therapies or interventions with novel mechanisms are required to overcome the tumor’s escape capabilities. An ideal therapeutic strategy should target multiple oncogenic pathways simultaneously or combine direct cytotoxicity with immune clearance to prevent residual resistant clones from causing tumor relapse (Natarelli et al., 2024). Additionally, the early elimination of micrometastases and continuous monitoring of dormant cancer cells are essential for improving long-term disease-free survival in melanoma patients. The suboptimal efficacy of current immunotherapies in immunologically “cold” tumors or in the presence of an immunosuppressive microenvironment underscores the urgent need for methods to reprogram the tumor microenvironment, enhancing tumor visibility and susceptibility to the immune system (Yang et al., 2023). Specifically, reducing intratumoral hypoxia, neutralizing the effects of immunosuppressive molecules, and activating host immune effector cells are necessary to maximize the efficacy of immunotherapies. The emergence of nanomedicine offers new hope for the treatment of melanoma. Nanomaterials possess unique advantages and can address the aforementioned challenges through synergistic strategies, thereby improving therapeutic efficacy and reducing side effects.

### 4.1 Unique advantages of nanodrug delivery

Nanocarriers, typically sized between 10 and 200 nm, can exploit the EPR effect of tumors to selectively accumulate at the tumor site (Mundra et al., 2015; Fang et al., 2020). This passive targeting mechanism increases local drug concentration in the tumor while reducing systemic exposure, thereby minimizing systemic toxicity. Compared to conventional drug formulations, nanodrugs can significantly enhance drug accumulation in the tumor while minimizing exposure in normal tissues (Mundra et al., 2015; Wang et al., 2022; Zeng et al., 2023). The encapsulation of drugs by nanomaterials also improves their pharmacokinetic properties. Nanodelivery systems notably enhance the aqueous solubility and stability of many anticancer drugs, prolong their circulation half-life, and optimize *in vivo* bioavailability (Zeng H. et al., 2022; Zeng H. et al., 2023). Lipophilic drugs delivered via nanoemulsions or liposomes can avoid rapid degradation or clearance in the bloodstream, allowing for a longer duration of action against tumors (Zeng H. et al., 2022). Additionally, the sustained-release characteristics of nanocarriers can prevent acute toxicity caused by high drug concentrations, achieving a more stable and prolonged therapeutic effect. Collectively, these features enhance therapeutic efficacy while reducing systemic side effects (Wang et al., 2022; Zeng H. et al., 2022; Zeng H. et al., 2023).

The surface of nanoparticles can be modified with ligands (such as antibody fragments, peptides, and sugar molecules) to endow them with the ability to actively recognize melanoma cells or their microenvironment (Moni et al., 2025). Conjugating integrin receptor ligands (such as RGD peptides) to the surface of nanocarriers enables specific binding to tumor vasculature and melanoma cells, thereby increasing the accumulation and cellular uptake of nanodrugs at the tumor site (Moni et al., 2025). This active targeting strategy further enhances the selectivity of treatment. Concurrently, some nanoparticles can be designed with ultra-small sizes or special shapes to facilitate deeper penetration into the tumor parenchyma, overcoming barriers posed by dense stroma and achieving uniform drug distribution in large-volume tumors. By combining active and passive targeting, nanodrug delivery holds promise to address the challenges of poor targeting and penetration associated with conventional drugs (Moni et al., 2025).

Nanocarriers can simultaneously deliver multiple therapeutic agents through physical encapsulation or chemical conjugation, enabling the integration of synergistic therapies on a single nanoplatform (Han et al., 2020; Liang et al., 2022; Zeng Z. et al., 2022; Moni et al., 2025). A single nanoparticle can co-deliver chemotherapeutic agents, photosensitizers, and immunomodulatory agents, thereby achieving a “one-stop” combination therapy. Compared to the conventional administration of different drugs separately, the nanoplatform ensures spatiotemporal synergistic delivery of the components: they are transported together to the tumor site and released in a designed ratio, thereby exerting synergistic effects within the same tumor cell or microenvironment. This synchronized drug delivery enhances the therapeutic efficacy and avoids the asynchrony caused by differences in the pharmacokinetics of individual drugs (Moni et al., 2025). Studies have demonstrated that the co-delivery of two or more drugs in the form of nanocomplexes achieves a stronger antitumor effect than sequential monotherapy (Buskaran et al., 2021; Yan et al., 2022; Li et al., 2023). A nano-prodrug that simultaneously releases the chemotherapeutic agent oxaliplatin and an IDO inhibitor can induce immunogenic cell death in tumors while alleviating tumor immunosuppression, thereby achieving a tumor growth inhibition effect more than twice that of either monotherapy (Yan et al., 2022).

**FIGURE 2:**
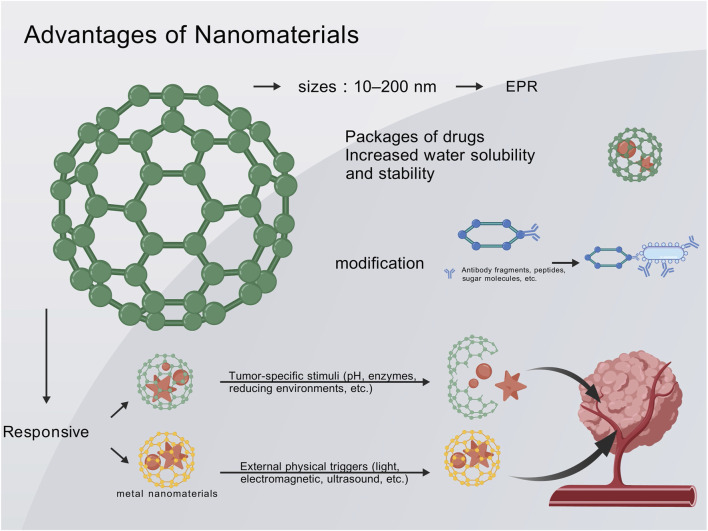
The unique advantages of nanodrug delivery.

Furthermore, through material design, nanocarriers can be engineered into “smart” systems that respond to tumor-specific stimuli (such as pH, enzymes, and reducing environments) or external physical triggers (Argenziano et al., 2022; Zeng Z. et al., 2022; Moni et al., 2025). PH-sensitive polymeric micelles disintegrate in the acidic tumor microenvironment, rapidly releasing the drug to attack adjacent tumor cells, while remaining stable in the neutral blood environment with minimal drug release (Zhao et al., 2019; Moni et al., 2025). Similarly, photothermal-responsive nanoparticles can locally heat up under near-infrared laser irradiation *in vitro*, triggering the instantaneous release of the loaded drug and achieving spatiotemporally controlled drug delivery (Zhao et al., 2019; Moni et al., 2025). These intelligent nanosystems ensure that the therapeutic effects are primarily localized to specific sites and times, further reducing damage to normal tissues and enhancing treatment precision. Through material modification and design, nanocarriers have achieved a series of improvements in stable drug transport, targeted release, and synergistic enhancement in melanoma therapy, providing technical support to overcome the limitations of conventional therapies (Figure 2).

### 4.2 Advantages of synergistic strategies

The core advantages of nanomaterials in the treatment of melanoma lie in enhanced targeting, overcoming biological barriers, and integrating multimodal therapies (Sang et al., 2019). These strengths directly address the bottlenecks in melanoma treatment. Nanodrug delivery can concentrate higher amounts of therapeutic agents at the tumor site, provide sustained and stable drug release, and integrate phototherapy and immunomodulation through a unified platform. The following sections will delve into several major synergistic treatment modalities and their mechanisms of action, elucidating how nanomaterials achieve optimal therapeutic effects at the molecular and cellular levels.

#### 4.2.1 Synergistic targeted delivery and immune modulation

Targeted drug delivery via nanocarriers enables the precise transportation of immune modulatory agents to the tumor and its microenvironment, thereby alleviating immune suppression and enhancing the host’s anticancer immune response. Simultaneously, the accumulation of nanocarriers at the tumor site increases the local concentration of tumor antigens or immune-stimulatory molecules. This synergistic combination of nanotargeting and immune modulation can enhance immune efficacy both locally within the tumor and systemically.

Nanomaterials can transport immune microenvironment modulators (such as small-molecule inhibitors, siRNA, and protein toxins) and selectively release them within the tumor to eliminate immune suppression and promote immune activation (Li and Burgess, 2020). Researchers have developed a nanoparticle that can simultaneously carry a mitochondrial respiration inhibitor and an MDSC inhibitor (Cabozantinib). This nanoparticle can reach melanoma lesions directly through a combination of intradermal and intravenous administration (Yang et al., 2023). This approach reverses the hypoxic and immunosuppressive states at the tumor site, reshapes the tumor immune microenvironment, and significantly amplifies the systemic antitumor immune effect (Yang et al., 2023).

Nanomaterials can also serve as vehicles to deliver tumor antigens and immune-stimulatory signals to lymph nodes, thereby activating the host immune system in a vaccine-like manner, while simultaneously releasing immune checkpoint inhibitors locally at the tumor site to relieve the tumor’s constraints on activated immunity (Yan et al., 2019). Encapsulating the oncolytic peptide LTX-315 and the immune adjuvant CpG in tumor-targeted polymeric nanoparticles and combining them with anti-PD-1 antibodies can induce a robust and long-lasting antitumor immune response in a melanoma mouse model (Zeng H. et al., 2022). Targeted nanocarriers ensure that immune-stimulatory molecules concentrate on hard-to-reach tumors and metastases, thereby generating a durable immune effect that is difficult to achieve with other administration methods.

**FIGURE 3:**
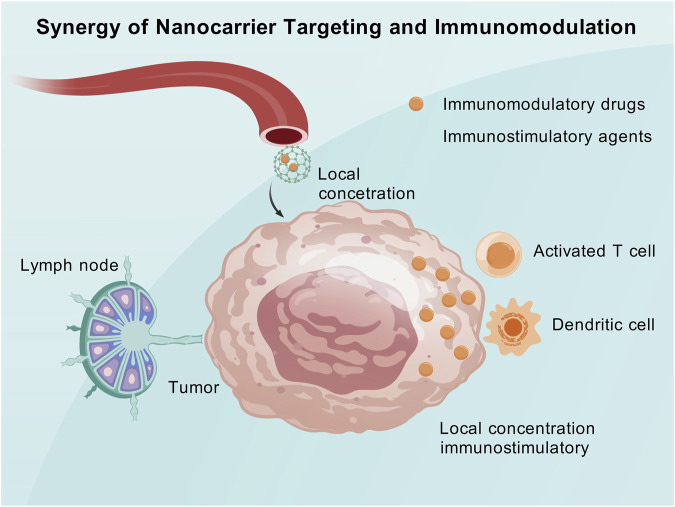
The synergy of targeted delivery and immune regulation.

The crux of the synergistic combination of targeted delivery and immune modulation lies in precise localization and a dual-pronged approach. Nanotechnology ensures that immune modulatory agents are released at the correct sites to exert maximal effects on tumor-associated immune cells. Simultaneously, by combining different immune mechanisms, a comprehensive enhancement of immune cytotoxicity is achieved (Atukorale et al., 2019). Compared to the traditional monotherapy with immune checkpoint inhibitors, the tumor microenvironment under nanosynergistic strategies is more conducive to immune cell infiltration and cytotoxicity, thereby converting “cold tumors” into “hot tumors” and improving the response rate to immunotherapy (Hanoteau et al., 2019). This mechanism holds significant clinical appeal, with the potential to address the issue of non-responsiveness to immunotherapy in many patients (Figure 3).

By taking advantage of their distinct physicochemical characteristics and biocompatibility, gold nanoparticles (AuNPs) have become a viable platform for improving the effectiveness of ICIs in melanoma prevention (Goddard et al., 2020; Alharbi et al., 2023). Low response rates and acquired resistance are two drawbacks of conventional immunotherapy that can be addressed with the conjugation of AuNPs with ICIs, such as anti-PD-1 or anti-PD-L1 antibodies (Wenthe et al., 2022; Alharbi et al., 2023). To achieve selective delivery to tumor sites, AuNPs can be functionalized with targeting ligands like aptamers, peptides, or antibodies. This reduces off-target effects and improves therapeutic precision (Goddard et al., 2020; Dykman et al., 2025). When it comes to melanoma, where the TME is frequently immunosuppressive, this targeted delivery is especially beneficial because it limits the effectiveness of ICIs (Wenthe et al., 2022).

AuNPs improve anti-melanoma immunity through a variety of mechanisms. First, by shielding ICIs from deterioration and extending their half-life, AuNPs can enhance their pharmacokinetics and bioavailability (Dykman et al., 2025). This is essential for maintaining immune activation over time and stopping tumor evasion. AuNPs can also alter the TME by encouraging the infiltration of NK cells and CTLs, both of which are critical for the destruction of tumor cells (Wenthe et al., 2022). A Th1-biased immune response, which is typified by the upregulation of interferon-γ (IFN-γ) and other pro-inflammatory cytokines, can be induced by AuNPs conjugated with ICIs, according to studies (Huang et al., 2020; Kesharwani et al., 2023). In addition to increasing the cytotoxicity of immune cells, this immune reprogramming also blocks immunosuppressive signals sent by myeloid-derived suppressor cells (MDSCs) and regulatory T cells (Tregs) (Alharbi et al., 2023).

Small molecule inhibitors of immune checkpoints, like BMS202, which targets the PD-1/PD-L1 axis, can be transported by AuNPs (Alharbi et al., 2023). By ensuring their controlled release at the tumor site, these inhibitors’ encapsulation within AuNPs maximizes their therapeutic effect while reducing systemic toxicity (Alharbi et al., 2023; Dykman et al., 2025). It has been demonstrated that nanodiamonds (NDs) loaded with BMS202 improve the interaction between peripheral blood mononuclear cells (PBMCs) and melanoma cells, resulting in notable anti-proliferative effects (Alharbi et al., 2023).

Combination therapies are another novel way that AuNPs are being used in melanoma immunotherapy. To enhance the anti-tumor immune response, AuNPs can be designed to co-deliver ICIs with other immunomodulatory substances, such as cytokines or Toll-like receptor (TLR) agonists (Huang et al., 2020; Kesharwani et al., 2023). It has been demonstrated that silica nanoparticles conjugated with TLR7 agonists increase T cell infiltration and upregulate IFN-γ expression, thereby improving the effectiveness of anti-PD-1 and anti-CTLA-4 antibodies (Huang et al., 2020). Tumor cells can undergo necroptosis when AuNPs functionalized with immunostimulatory ligands are present, releasing damage-associated molecular patterns (DAMPs) that further stimulate the immune system (Cai et al., 2023; Kesharwani et al., 2023).

AuNPs’ photothermal characteristics increase their usefulness in melanoma immunotherapy. AuNPs can cause localized hyperthermia in response to NIR light, which kills tumor cells directly and facilitates the entry of ICIs into the tumor tissue (Olesiak-Banska et al., 2019; Kesharwani et al., 2023). In order to prime the immune system for a stronger anti-tumor response, this photothermal effect can also cause ICD, which is characterized by the release of tumor antigens and the activation of DCs (Olesiak-Banska et al., 2019; Kesharwani et al., 2023) AuNPs’ optical characteristics allow for imaging and therapeutic response monitoring, offering a theranostic platform for individualized melanoma treatment (Goddard et al., 2020; Dykman et al., 2025).

Notwithstanding these developments, there are still obstacles in the way of AuNP-based immunotherapies’ clinical translation. It is necessary to address concerns like possible toxicity, adverse events linked to the immune system, and the difficulty of large-scale production (Kesharwani et al., 2023; Dykman et al., 2025). In order to improve AuNPs’ safety and effectiveness, research is still being done to optimize their size, shape, and surface chemistry (Goddard et al., 2020; Dykman et al., 2025). The development of predictive biomarkers and advanced delivery systems will be crucial for identifying patients who are most likely to benefit from AuNP-conjugated ICIs (Huang et al., 2020; Wenthe et al., 2022). A novel strategy for boosting anti-melanoma immunity is the conjugation of immune checkpoint inhibitors with gold nanoparticles. AuNPs can enhance ICI delivery, efficacy, and safety by utilizing their special qualities. This also makes combination therapies and theranostic applications possible. AuNP-based immunotherapies have great potential to overcome the drawbacks of existing treatments and enhance the prognosis of melanoma patients as this field of study develops (Wenthe et al., 2022; Alharbi et al., 2023).

#### 4.2.2 Synergistic effects of PDT and immunotherapy

Photodynamic therapy (PDT), leveraging nanomaterials to convert light energy into cytotoxic effects, achieve synergistic phototherapy and immunotherapy. This combination enables the simultaneous ablation of localized tumors and systemic immune clearance, thereby fulfilling the dual objectives of controlling both primary and metastatic lesions (Lopes et al., 2022a; Yan et al., 2022; Zhao et al., 2022). PDT employs nanocarriers to deliver photosensitizers that accumulate within tumors. Upon irradiation with specific wavelengths of light, reactive oxygen species (ROS) are generated, inducing tumor cell death and an inflammatory response (Liu P. et al., 2019). This process releases a large amount of tumor antigens, rendering the tumor visible to the immune system. When combined with immune checkpoint blockade, the immunogenic effects triggered by PDT can be significantly enhanced (Liu H. et al., 2019). Recently, a study designed a matrix metalloproteinase (MMP)-responsive nanoparticle co-loading the photosensitizer indocyanine green (ICG) and anti-PD-L1 antibody. Following light irradiation, PDT-induced tumor cell death releases antigens, sensitizing residual tumors to PD-1 antibody therapy. The combination therapy achieved a 73.2% reduction in tumor growth (Yan et al., 2022). Another nanoplatform co-delivered a photosensitizer with PD-L1 siRNA. The ROS generated by PDT promote DC antigen uptake, while siRNA downregulates PD-L1 expression in tumor cells. The synergistic therapy completely eradicated tumors in a mouse melanoma model without significant toxicity (Yan et al., 2022). These results demonstrate that PDT provides tumor-associated antigens required by the immune system, while checkpoint inhibition alleviates T-cell dysfunction. Together, they induce a systemic antitumor immune response through nanocarriers in the same spatiotemporal context.

Using the distinct mechanisms of both modalities to boost anti-tumor immunity and enhance clinical outcomes, the synergistic effects of PDT and immunotherapy in the treatment of melanoma represent a revolutionary approach in oncology. Because of its hypoxic microenvironment and immune evasion tactics, melanoma, an aggressive and extremely deadly cancer, frequently defies standard treatments (Pires et al., 2020). This minimally invasive procedure uses photosensitizers that are triggered by particular light wavelengths to produce cytotoxic reactive oxygen species (ROS), which kill tumor cells directly and cause ICD (Lamberti et al., 2019; Sasaki et al., 2023). Calreticulin, ATP, and HMGB1 are examples of damage-associated molecular patterns (DAMPs) released during ICD, which stimulate DCs and encourage CTL infiltration into the TME (Ji et al., 2022; Sasaki et al., 2023). The immunosuppressive TME and the insufficient activation of adaptive immunity frequently limit the effectiveness of PDT alone (Pires et al., 2020; Ji et al., 2022).

Immunotherapy has transformed the treatment of melanoma by reestablishing T cell-mediated anti-tumor responses, especially ICIs that target the PD-1/PD-L1 and CTLA-4 pathways (Gu et al., 2022; Ji et al., 2022). The absence of tumor immunogenicity and the existence of immune-suppressive factors in the TME contribute to the suboptimal response rates to ICIs (Ji et al., 2022). By increasing tumor immunogenicity and boosting the immune response, PDT in conjunction with immunotherapy overcomes these drawbacks. PDT increases tumor cells’ expression of immune co-stimulatory molecules like CD80, which improves CD28-mediated T cell activation and works in concert with anti-CTLA-4 treatment (Gu et al., 2022). Anti-PD-1/PD-L1 antibodies can also target PDT-induced upregulation of PD-L1 on tumor cells, strengthening the immune response against tumors (Gu et al., 2022; Ji et al., 2022). In addition to enhancing local tumor control, this combination also produces systemic anti-tumor immunity, which includes abscopal effects, in which systemic immune responses cause untreated metastatic lesions to regress (Gu et al., 2022; Sasaki et al., 2023).

The potential of this combinatorial approach in melanoma models has been shown in recent studies. In murine melanoma models, the combination of PDT and anti-PD-1 therapy dramatically reduced tumor growth and extended survival; in certain instances, total tumor regression was seen (Gu et al., 2022; Sasaki et al., 2023). Using photosensitizers based on nanoparticles, like Photodithazine, has demonstrated promise in increasing the effectiveness of PDT by enhancing light penetration and tumor targeting, especially in pigmented melanomas (Pires et al., 2020). These developments demonstrate how PDT can transform “cold” tumors, which have low immune infiltration, into “hot” tumors, which have strong immune activation, increasing the effectiveness of immunotherapy (Lamberti et al., 2019; Ji et al., 2022). The hypoxic TME, a melanoma hallmark, presents additional difficulties that are addressed by combining PDT with immunotherapy. Hypoxia increases PD-L1 expression and attracts immunosuppressive cells, which both enhance immune suppression and lessen the effectiveness of PDT by reducing ROS generation (Pires et al., 2020; Ji et al., 2022). It has been demonstrated that techniques like vascular normalization with tyrosine kinase inhibitors like Lenvatinib reduce hypoxia, increase PDT effectiveness, and boost CTL infiltration, all of which improve the TME for immunotherapy (Ji et al., 2022; Zheng et al., 2023). New possibilities to overcome hypoxia-related resistance and improve the synergistic effects of PDT and immunotherapy are presented by the development of novel photosensitizers with enhanced oxygen-independent mechanisms, such as type I PDT (Li et al., 2025). Utilizing the complementary mechanisms of both modalities, PDT and immunotherapy together offer a promising approach to treating melanoma by increasing tumor immunogenicity, overcoming immune suppression, and triggering systemic anti-tumor immunity. Advanced photosensitizers, vascular normalization techniques, and immune checkpoint blockade combined have the potential to revolutionize melanoma treatment and provide hope for better results in this difficult condition. To further increase the effectiveness of this synergistic approach, future research should concentrate on developing new combinatorial techniques, identifying predictive biomarkers, and optimizing treatment protocols (Pires et al., 2020; Sasaki et al., 2023).

#### 4.2.3 Synergistic effects of PTT and immunotherapy

The combination of immunotherapy and PTT has shown promise in treating melanoma by utilizing the complementary mechanisms of both treatments to improve therapeutic results. PTT releases tumor-associated antigens (TAAs) and damage-associated molecular patterns (DAMPs), which promote DC maturation and antigen presentation. It also causes tumor cell ablation and ICD by using light-absorbing agents to create localized hyperthermia (Liu Y. et al., 2019; Xiong et al., 2023; Jiang et al., 2025). By priming the immune system, this procedure makes it possible for immunotherapy to boost anti-tumor immune responses. In addition to targeting the primary tumor, PTT and immunotherapy also create systemic anti-tumor immunity, which may help treat metastatic lesions by means of the abscopal effect (Kim S. et al., 2021; Nam et al., 2021).

**FIGURE 4:**
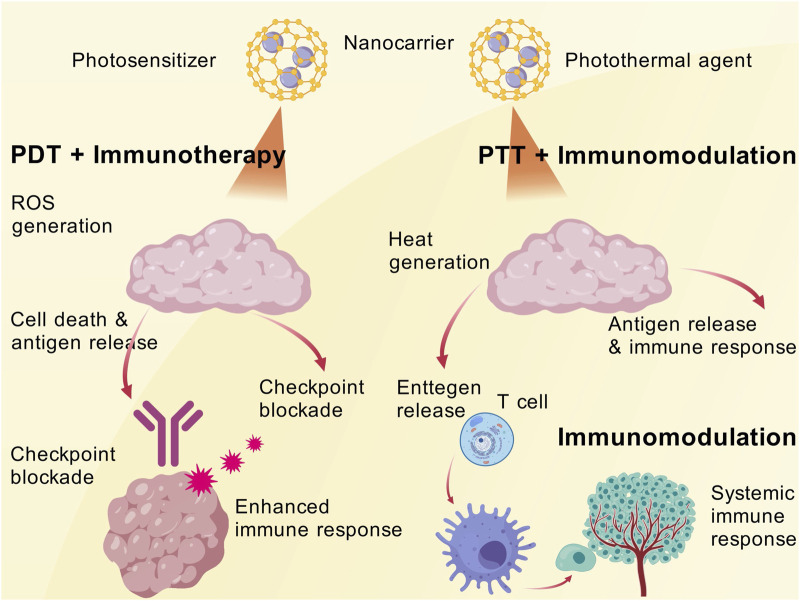
The synergy of PDT/PTT and immunity.

PTT employs nanoscale photothermal agents to convert light energy into heat, thereby locally ablating cancer cells (Yu et al., 2022). The introduction of immune adjuvants or pro-inflammatory factors during PTT can further enhance the immune system’s response to tumor antigens (Miele et al., 2022). One study constructed a biomimetic nanoplatform using synthetic high-density lipoprotein (sHDL) as a carrier to load photothermal agents and immune adjuvants. The danger signals generated by photothermal ablation of tumors, combined with adjuvant stimulation, significantly activated DCs and CTLs. The synergistic therapy not only inhibited the growth of primary tumors but also effectively prevented tumor recurrence (Yan et al., 2022). The notable advantage of synergistic phototherapy and immunotherapy lies in the transformation of localized treatment into systemic efficacy, achieving a dual impact on both overt tumors and occult lesions. The utilization of nanomaterials enhances the accumulation of photosensitizers/photothermal agents within tumors, ensuring adequate photodestruction, and allows for the concurrent delivery of immune modulatory components, ensuring timely and robust immune activation. For melanoma, a highly metastatic disease, photothermal immunotherapy has the potential to eliminate microscopic metastases that are difficult to reach surgically, thereby reducing the recurrence rate. Numerous preclinical studies have already validated the effects of phototherapy combined with checkpoint inhibitors or adjuvants, and the mechanistic breakthroughs offer a novel comprehensive treatment strategy for melanoma (Figure 4).

The ability of PTT to alter the TME, which is frequently immunosuppressive and resistant to immunotherapy, is one of the main advancements in this combination therapy. By attracting neutrophils, stimulating NK cells, and improving DC function, PTT can debulk large tumors, lower physical barriers within the TME, and change the immune landscape (Kim S. et al., 2021; Nam et al., 2021). PTT’s effectiveness may be limited by its ability to produce short-term immunosuppressive effects, such as the enrichment of regulatory T cells (Tregs) and M2-like macrophages (Nam et al., 2021). By encouraging the infiltration and activation of cytotoxic CD8^+^ T cells, decreasing Treg populations, and causing the TME to change toward a more immunogenic state, immunotherapy counteracts these effects (Kim S. et al., 2021; Nam et al., 2021). The potential of this strategy for advanced melanoma has been highlighted by studies showing that PTT in conjunction with neoantigen cancer vaccines can completely eradicate large primary tumors and have strong abscopal effects against distant metastases (Nam et al., 2021). To maximize synergism, PTT and immunotherapy timing and sequence are crucial. According to preclinical models, immunotherapy after PTT improves the recruitment and activation of T cells specific to tumors, resulting in long-lasting anti-tumor responses (Kim S. et al., 2021; Nam et al., 2021). This sequence supports the idea that immunotherapy maintains and strengthens the immune response, while PTT-induced ICD primes the immune system. The therapeutic index is further improved by the precise control of treatment parameters, such as drug delivery, heat distribution, and light dosage, made possible by the use of nanotechnology in PTT, such as engineered nanoparticles or photothermal agents (Liu Y. et al., 2019; Jiang et al., 2025). In preclinical melanoma models, multifunctional nanocarriers that combine PTT with immune adjuvants or checkpoint inhibitors have demonstrated superior efficacy, resulting in synergistic tumor regression and extended survival (Du et al., 2017; Liu et al., 2023).

The capacity of nanoparticles to alter the tumor microenvironment enhances the synergistic effects of PTT and ICB. Because of their superior surface plasmon resonance, biocompatibility, and photothermal conversion efficiency, gold nanoparticles (AuNPs) have been used extensively in PTT (Yang et al., 2019). Through the EPR effect, these nanoparticles can accumulate in tumor tissues, ensuring localized heat generation and reducing harm to nearby healthy tissues (Ding et al., 2024). The heat produced by AuNPs enhances the antitumor immune response by inducing ICD and encouraging the release of pro-inflammatory cytokines (Ding et al., 2024). It has been demonstrated that PTT and ICB together enhance the expression of MHC class I and co-stimulatory molecules on tumor cells, increasing their immunogenicity and vulnerability to T-cell-mediated destruction (Ledezma et al., 2020).

Using gold nanorods and anti-PD-1 peptides in microneedle drug delivery systems is another creative strategy. By penetrating the skin and delivering therapeutic agents straight to the tumor site, these systems can increase T lymphocytes’ capacity for cytotoxicity (Weng et al., 2023). The gold nanorods produce heat when exposed to NIR light, which helps to further ablate tumors. This localized delivery approach is a promising melanoma treatment strategy because it lowers systemic toxicity while simultaneously increasing treatment efficacy (Weng et al., 2023).

The immunogenicity of melanoma cells may be increased by combining PTT with epigenetic therapy. Poly (lactic-co-glycolic) acid (PLGA) nanoparticles that co-encapsulate Nexturastat A (NextA) and ICG have been created to administer photothermal and epigenetic treatments (Ledezma et al., 2020). ICG, a photothermal agent, produces heat when exposed to NIR laser light, whereas NextA, an epigenetic medication, increases the expression of MHC class I and tumor antigens on melanoma cells by blocking histone deacetylase (HDAC) activity (Ledezma et al., 2020). It has also been investigated to combine ICB with upconversion nanoparticles (UCNPs). Deeper tissue penetration and more potent phototherapy are made possible by UCNPs’ ability to transform NIR light into visible or ultraviolet light (Wang et al., 2019). These nanoparticles can produce synergistic photothermal and photodynamic effects when functionalized with ICG and rose bengal (RB), which will further improve ICD and TAA release (Wang et al., 2019). Antigen-presenting cells subsequently present the captured TAAs, triggering a strong immune response that is specific to the tumor. In preclinical models, this strategy has demonstrated great promise in inhibiting primary and distant tumors, especially when paired with anti-CTLA-4 therapy (Wang et al., 2019).

Despite the encouraging outcomes, there are still obstacles to overcome before this combination therapy can be used in clinical settings. Treatment protocols must be carefully optimized due to the heterogeneity of melanoma, variations in immune responses, and the possible toxicity of combination treatments. The safety and effectiveness of PTT in combination with ICIs or other immunotherapeutic agents are being investigated in ongoing clinical trials; initial findings point to higher response rates and better survival outcomes (Kim S. et al., 2021; Phadke et al., 2021). By using cutting-edge imaging methods like photoacoustic imaging (PAI), PTT delivery may be guided and treatment responses can be tracked in real-time, improving accuracy and minimizing off-target effects (Liu Y. et al., 2019). PTT and immunotherapy work in concert to treat melanoma in a way that is revolutionary since it targets both systemic immune activation and local tumor management. This combination therapy may be able to overcome resistance mechanisms and enhance outcomes for patients with advanced melanoma by utilizing the immunogenic qualities of PTT and the immune-enhancing potential of immunotherapy.

#### 4.2.4 Synergy of dynamic monitoring and precision intervention

Nanotechnology also supports real-time monitoring and dynamic adjustment during the therapeutic process, achieving theranostics integration. Through the synergy of diagnosis and therapy, clinicians can intervene precisely, significantly enhancing the safety and efficacy of treatment (Qin et al., 2019).

Traditionally, therapeutic efficacy is often assessed weeks after treatment through imaging or biochemical markers, which fails to provide timely insights into early treatment responses. To address this, researchers have developed “self-reporting” nanodrugs that emit detectable signals upon drug release or encounter with tumor enzymes, enabling real-time visualization of therapeutic efficacy (Kulkarni et al., 2016). An experiment designed a two-in-1 reporting nanoparticle that, on one hand, delivers chemotherapeutic or immunotherapeutic agents to the tumor and, on the other hand, emits fluorescence/magnetic resonance signals to indicate the degree of drug release and cell killing (Kulkarni et al., 2016). Using such nanoprobes, researchers can differentiate whether the tumor is responsive to therapy early after administration, thereby allowing timely adjustments to the treatment plan and avoiding the time wastage and side effects associated with ineffective therapy (Kulkarni et al., 2016). Experiments have demonstrated that these nanoprobes can directly report the *in vivo* activity of anticancer drugs, promptly identifying responders and non-responders, and effectively improving treatment outcomes in mouse models. This heralds a future clinical scenario where physicians can leverage similar nanoprobes to monitor real-time responses of melanoma patients to therapy and make personalized adjustments.

The aforementioned stimulus-responsive nanocarriers not only enhance targeting but also provide a means for precision intervention (Zhao et al., 2021; Tewari et al., 2022). Upon reaching the tumor, these responsive nanomaterials are triggered by the microenvironment to release drugs that kill tumor cells (Tewari et al., 2022; Moni et al., 2025). This on-demand release ensures that the intervention is highly precise in both time and space. When combined with real-time detection technologies, the future potential includes: nanoprobes monitoring changes in tumor biomarkers (Wang et al., 2016), and instantaneously triggering the nanosystem to release additional doses of drugs or signaling molecules for intervention at the very onset of tumor relapse. This autonomous responsiveness and regulatory capability transforms treatment from a passive to an actively controllable process (Liang et al., 2020). A recent study utilized a blue-light-induced CRISPR nanosystem to achieve precise spatiotemporal gene-editing intervention in melanoma: only when needed, light irradiation of the skin triggers the expression of CRISPR/Cas9 within the nanocarrier to excise oncogenes, thereby inhibiting tumor growth (Wu et al., 2020). This approach, which integrates gene therapy with external physical control, demonstrates the potential of precision intervention (Wu et al., 2020).

**FIGURE 5:**
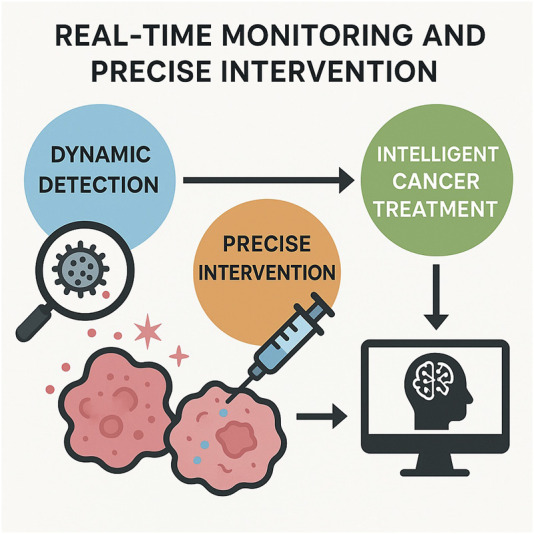
The synergy of dynamic monitoring and precision intervention.

The significance of the synergy between dynamic monitoring and precision intervention lies in enhancing the real-time nature and personalization of treatment. Nanotheranostic platforms provide clinicians with visual means to monitor the therapeutic process in real-time and make immediate corrections when deviations from the desired trajectory occur. Meanwhile, responsive nanosystems ensure that drugs act at the correct site and time, minimizing randomness. For rapidly mutating and progressing tumors like melanoma, this timeliness is crucial. Real-time monitoring can detect the emergence of drug resistance or metastatic lesions early and provide immediate additional interventions, thereby increasing the success rate of treatment. Precision intervention reduces unnecessary systemic toxicity and improves the quality of life for patients. It can be anticipated that with the development of nanosensing and controlled-release technologies, the synergy of “treat, diagnose, and adjust” will become possible, ushering in an era of intelligent melanoma therapy (Figure 5).

#### 4.2.5 Other emerging synergistic therapeutic approaches

Beyond the aforementioned primary types, nanomaterials also provide a platform for a variety of innovative synergistic therapeutic approaches. Nanoparticles can combine chemotherapeutic agents with photosensitizers or photothermal agents to achieve “light-controlled chemotherapy”. Upon light irradiation, the photosensitizer or photothermal agent directly kills cells while simultaneously promoting the localized release of chemotherapeutic drugs from the nanocarrier (Wang et al., 2018). The combination of these two modalities significantly enhances the cytotoxicity against tumor cells (Kim et al., 2023). This approach is particularly suitable for superficial and visible melanoma lesions, where local irradiation can achieve high-concentration, on-demand drug release and deep penetration. It may potentially overcome the limitations of traditional chemotherapy in treating melanoma.

Metal nanoparticles with high atomic numbers serve as potent radiosensitizers, enhancing the damage to tumors by ionizing radiation. When these nanoparticles are delivered into melanoma cells followed by radiotherapy, they can increase the production of reactive oxygen species and the number of DNA double-strand breaks, thereby improving the efficacy of radiotherapy (Bemidinezhad et al., 2024). Some novel nanoparticles can simultaneously trigger both radiotherapy and photodynamic therapy under irradiation, while also inducing immune responses through the release of tumor antigens (Zhang et al., 2020). Experiments have shown that these nanoparticles can enhance radiotherapy, induce tumor oxidative damage, and stimulate antitumor immunity in melanoma models, significantly improving overall therapeutic outcomes. Additionally, the combination of radiotherapy, nanoparticles, and immunotherapy has been proposed to integrate local and systemic control of refractory melanoma (Bemidinezhad et al., 2024).

Nanomaterials can serve as carriers for cancer vaccines, delivering melanoma antigens and adjuvants to elicit an immune response within the body, and can be combined with cell therapies to enhance their efficacy (Chu et al., 2022). Melanoma cell lysates or peptide antigens loaded onto nanoparticles or hydrogels, administered together with immune modulators, can play a role in preventing recurrence after surgery (Yan et al., 2022). One study encapsulated tumor cells and the PD-L1 inhibitor JQ1 into a hydrogel and triggered antigen and drug release via PDT, resulting in a significant increase in DC maturation rate in lymph nodes (up to 62%) and effectively inhibiting postoperative autologous tumor recurrence and metastasis (Yan et al., 2022). Additionally, nanocarriers can be used for the delivery of gene therapies (such as siRNA, miRNA, or CRISPR components) to directly target and silence oncogenes or reverse resistance mechanisms, working synergistically with conventional treatments. Cationic lipid nanoparticles delivering CRISPR/Cas9 ribonucleoproteins to specifically knock out the BRAF V600E oncogenic mutation in melanoma can induce tumor cell death and enhance sensitivity to other drugs (Yang et al., 2024). In the future, such gene-editing therapies are expected to be combined with immunotherapy to form new synergistic modalities.

In recent years, the continuous emergence of various synergistic approaches reflects the concept of integrating multiple therapies on a nanoplatform to achieve comprehensive treatment. Whether it is the combination of physical therapies (light, radiation, heat) with chemical and biological therapies, or the integration of cutting-edge genetic engineering with immunotherapy, nanomaterials have provided the required multifunctional carriers and spatial convergence mechanisms. By targeting tumor cells with a combination of methods, nanosystems overcome the blind spots of monotherapy. These emerging synergistic mechanisms have demonstrated effective antitumor effects at the experimental level and are expected to further enrich the methods of comprehensive melanoma treatment (Table 1).

**TABLE 1:** Nanomaterials in synergistic therapy of melanoma.

Materials	Mechanism of action	Synergistic strategy	Experimental model	Therapeutic efficacy	Ref.
MS-275 (HDAC inhibitor), V-9302 (glutamine metabolism inhibitor), ROS-responsive nanoparticles	Induction of ROS storm and pyroptosis by inhibiting the mTOR pathway and reducing GSH levels, enhancing immune cell infiltration, and converting “immune-cold” tumors to “immune-hot” tumors	Co-delivery of MS-275 and V-9302 using ROS-responsive nanoparticles to synergistically induce ROS storm and pyroptosis	*In vitro*: OCM-1, MUM-2B, and 92.1 cell lines; *In vivo*: BALB/c nude mouse model with OCM-1 cells, C57BL/6 mouse model with B16-F10 cells	Significant inhibition of cell proliferation and induction of pyroptosis *in vitro*; marked tumor growth inhibition and enhanced anti-tumor immunity *in vivo*, with long-term immune memory when combined with *α*-PD-1	Ren et al. (2024a)
CoFe2−xDyxO4 nanoparticles, hydroxypropyl-γ-cyclodextrin (HPGCD)	Disruption of the mTOR pathway and glutathione metabolism, leading to apoptosis	Dysprosium (Dy)-doped cobalt ferrite (CoFe2O4) nanoparticles synthesized by combustion and coated with HPGCD to enhance dispersion and biocompatibility	*In vitro*: A375 melanoma cells, MCF-7 breast cancer cells, and HaCaT normal keratinocytes	Significant cytotoxicity in melanoma and breast cancer cells with minimal effects on normal keratinocytes; apoptosis confirmed by nuclear fragmentation, cell shrinkage, and changes in protein expression levels of caspase-9, p53, and cyclin D1	Rotunjanu et al. (2023)
Various nanocarriers (liposomes, niosomes, transferosomes, ethosomes, cubosomes, dendrimers, cyclodextrins, solid lipid nanoparticles, carbon nanotubes)	Enhanced skin penetration, stability, and bioavailability of drugs through various nanocarriers, increasing drug accumulation in tumor cells and inhibiting proliferation and metastasis	Development of different nanocarriers with surface modifications and targeting ligands to improve drug delivery efficiency	*In vitro*: melanoma cell lines (A375, B16F10); *In vivo*: Mouse models	Multiple nanocarriers demonstrated inhibitory effects on melanoma cell proliferation *in vitro*, with some showing significant tumor growth inhibition and reduced lung metastasis *in vivo*, improved skin penetration, and enhanced drug accumulation in tumor cells	Saeidi et al. (2023)
Gold nanostars (Au NSs), thiol-modified polyethylene glycol (PEG-SH)	Photothermal conversion of near-infrared (NIR) laser energy into heat by Au NSs, leading to cancer cell death through apoptosis and photothermolysis	Utilization of the intercellular trafficking capability of uveal melanoma cells to redistribute Au NSs between cells, enhancing photothermal therapy efficiency	*In vitro*: Uveal melanoma cells; Laser: 800 nm Ti:sapphire femtosecond pulse laser	Pulse laser High photothermal therapy (PPTT) efficiency achieved after multiple cell division cycles with low initial Au NS concentrations (2 pM and 8 pM). Effective PPTT observed even after mixing Au NS-loaded and non-loaded cells, maintaining photothermal properties through several division cycles. Potential for co-therapy to disperse plasmonic gold nanostructures across affected tissues, increasing the effectiveness of classic PPTT	Ahijado-Guzmán et al. (2020)
Amino-functionalized mesoporous silica nanoparticles (MSN-NH_2_)	MSN-NH2 enhances drug adsorption and loading capacity through amino functionalization, particularly for 5-FU and DEX.	Combination of 5-FU and DEX delivered via MSN-NH2 to enhance antitumor efficacy against melanoma	HT-144 melanoma cell line	MSN-NH2 exhibited no toxicity to melanoma cells but significantly enhanced the cytotoxicity of 5-FU and DEX when loaded with drugs	Almomen et al. (2020)
Prussian blue nanoparticles (PBNPs)	PBNP-PTT (photothermal therapy) induces immunogenic cell death (ICD) in melanoma cells, releasing tumor antigens and upregulating markers associated with antigen presentation and immune cell co-stimulation (e.g., CD80, CD86, MHC-I, CD137L)	PBNP-PTT provides local tumor ablation and immunogenicity, releasing tumor antigens that prime the immune system	*In vitro* studies using murine SM1 and human WM9/WM793 melanoma cell lines	PBNP-PTT alone eliminated primary tumors but did not generate long-term immunological memory or eliminate distal tumors	Balakrishnan et al. (2022)
Polyethylene glycol (PEG)-modified doxorubicin (DOX) encapsulated nanoparticles (DOX-NP)	WEC treatment enhances the enhanced permeability and retention (EPR) effect in tumors, leading to increased intratumor accumulation of DOX-NP.	Combination of DOX-NP with WEC leverages the enhanced intratumor delivery of nanoparticles and direct anticancer activity of WEC.	*In vitro* studies using B16-F1 murine melanoma cells	WEC treatment resulted in a 2.3-fold higher intratumor accumulation of DOX-NP compared to untreated controls	Khatun et al. (2022)
Bovine serum albumin (BSA)-coated silver nanoparticles (BSA-Silver NPs)	BSA-Silver NPs induce cytotoxic effects on B16F10 melanoma cells through oxidative stress-mediated reactive oxygen species (ROS) generation	Combination of BSA-Silver NPs with photothermal therapy leverages the cytotoxic effects of ROS generation and hyperthermia-induced cell death	*In vitro* studies using B16F10 murine melanoma cells	BSA-Silver NPs showed significant cytotoxicity on B16F10 cells with an IC50 value of 65.8 ± 9.2 µM	Kim et al. (2021a)
Pluronic^®^ F127 copolymer	Biotin, spermine, and folic acid are covalently linked to the F127 copolymer to form targeted micelles	Combination of biotin, spermine, and folic acid with the F127 copolymer micelles leverages their targeting capabilities to improve HY delivery to melanoma cells	*In vitro* studies using B16F10 murine melanoma cells	HY-loaded F127-BT, F127-SN, and F127-FA micelles showed enhanced photodynamic activity against B16F10 cells compared to non-modified F127 micelles	De Morais et al. (2022)
Genipin	Genipin crosslinks with polyamines to form cationic polymers	Adjusting the glycine-to-genipin molar ratio to optimize polymer size, zeta potential, and siRNA complexation ability	B16F10 murine melanoma cells	Efficient siRNA delivery to B16F10 cells with up to 60% gene knockdown efficiency	Della Pelle et al. (2024)
Hollow mesoporous copper sulfide (CuS) nanoparticles	CuS NPs serve as carriers for PMS and generate heat upon NIR-II laser irradiation (1064 nm)	NIR-II laser irradiation (1064 nm) provides deep tissue penetration and efficient photothermal conversion	*In vitro* studies using B16F10 murine melanoma cells	Efficient production of sulfate radicals and hydroxyl radicals in an oxygen-independent manner, overcoming limitations of hypoxia and low H_2_O_2_ content in tumor microenvironments	Ding et al. (2022)
Gold nanoparticles (AuNPs) coated with a mixture of hyaluronic acid (HA) and oleic acid (OA)	AuNPs absorb NIR light and convert it into heat, causing local hyperthermia	NIR laser irradiation enhances the photothermal effect of AuNPs	*In vitro* studies: Murine B16F10 and human A375 melanoma cell lines	Combined therapy significantly reduced cell viability in both B16F10 and A375 cell lines, with a pronounced effect at higher AuNP concentrations	Lopes et al. (2025)
Copper oxide nanoparticles (CuO NPs)	ES@CuO NPs release Cu^2+^ and ES in the acidic tumor microenvironment	ES@CuO NPs trigger cuproptosis, which enhances the release of DAMPs and promotes lymphocyte infiltration	*In vitro*: Murine B16 melanoma cell line	ES@CuO NPs alone significantly inhibited tumor growth in B16 melanoma cells and promoted cuproptosis *in vitro* and *in vivo*	Lu et al. (2024)
Gadolinium-DOTA (Gd-DOTA)	Gd-DOTA/DOX-loaded nanodroplets (NDs) encapsulate DOX and Gd for MRI-guided drug delivery	Ultrasound exposure enhances the release of DOX from NDs, increasing drug concentration at the tumor site	*In vitro*: B16F10 melanoma cancer cells and L929 normal fibroblast cells	Gd-DOTA/DOX NDs exhibited high stability and biocompatibility *in vitro* and *in vivo*	Maghsoudinia et al. (2022)
Six melanoma helper peptides (6MHP)	Nanoliposomes encapsulate melanoma helper peptides and KDO2 to enhance immune responses	Co-delivery of peptides and KDO2 in a single nanoliposome formulation ensures simultaneous delivery to APCs	*In vitro*: Peripheral blood mononuclear cells (PBMC) and sentinel immunized nodes (SIN) from melanoma patients	Nanoliposomes demonstrated stable size distribution and high encapsulation efficiency for all six peptides	Salotto et al. (2022)
Cholic acid-poly (lactide-co-glycolide)-b-polyethylene glycol (CA-PLGA-b-PEG)	AS1411 aptamer binds specifically to nucleolin, which is overexpressed on the surface of malignant melanoma cells, enabling active targeting of DTIC-loaded nanoparticles to tumor cells	AS1411 modification on nanoparticles enhances specific uptake by tumor cells expressing nucleolin, improving the therapeutic index	Human melanoma A875 cells	DTIC-NPs-Apt exhibited the strongest cytotoxicity against A875 cells, with an IC50 value less than half of that of DTIC-NPs. The viability of A875 cells treated with DTIC-NPs-Apt was significantly lower than that treated with free DTIC or DTIC-NPs	Xiong et al. (2022)
Poly (N-vinylcaprolactam) (PVCL) nanogels (NGs)	The PVCL NGs are crosslinked by disulfide bonds, which are cleaved in the presence of high glutathione (GSH) levels in the tumor microenvironment, leading to the release of Mn2+ ions for enhanced T1-weighted MRI and DOX for chemotherapy.v	The NGs integrate diagnostic (T1-weighted MRI) and therapeutic (chemotherapy) functions in a single platform	B16 melanoma cells were used to evaluate the cytotoxicity, cellular uptake, and redox-responsive release of DOX/MnO2@PVCL NGs	DOX/MnO2@PVCL NGs showed significantly enhanced cytotoxicity against B16 cells compared to free DOX, especially when combined with UTMD. Cellular uptake studies demonstrated that UTMD significantly increased the internalization of NGs by tumor cells	Xu et al. (2020)
Transdermal photothermal nanosensitizer (FSGG) loading Gal-9 siRNA (FSGG/siGal-9	GNR-MUA converts NIR light into heat, causing localized hyperthermia to destroy tumor cells	The synergistic effects of photothermal therapy and Gal-9 silencing enhance anti-tumor efficacy	B16-F10 murine melanoma cells	FSGG/siGal-9 significantly reduced Gal-9 expression in B16-F10 cells, enhanced the photothermal killing of tumor cells, and promoted T cell proliferation and cytotoxicity	Ren et al. (2024b)

Theranostic nanoparticles and other multifunctional nanomaterials that combine therapeutic and diagnostic properties are revolutionary in contemporary medicine, especially when it comes to the treatment of cancer. By combining targeted therapeutic delivery with cutting-edge imaging modalities, these nanoparticles allow for the simultaneous diagnosis and treatment of conditions like melanoma. In order to create a dual-purpose platform for MRIDTIC and photothermal imaging (PI), manganese-doped mesoporous silica nanoparticles (MSN(Mn)) have been engineered to incorporate chemotherapeutic agents like dacarbazine (DTIC) and photothermal agents like ICG (Zhang et al., 2021). This integration greatly improves the precision and efficacy of melanoma treatment by enabling accurate tumor localization and real-time therapeutic efficacy monitoring. The MSN(Mn)-ICG/DTIC system showed outstanding photothermal heating capabilities, high drug-loading efficiency, and superior biocompatibility, all of which add to its strong antitumor effects *in vivo* and *in vitro* (Zhang et al., 2021). By using the special physicochemical characteristics of nanomaterials to achieve localized and controlled drug release, these innovations overcome the drawbacks of conventional therapies, such as poor target specificity and systemic toxicity (Nima et al., 2019; Zhang et al., 2021).

Superparamagnetic iron oxide or gold nanoparticles, which function as MRI contrast agents or photothermal ablation tools, respectively, are examples of inorganic cores with exploitable physical properties that are frequently incorporated into theranostic nanoparticle designs (Mikhaylov et al., 2022; Lin and Zhang, 2023). To improve biocompatibility and immunological stealth, these cores are usually coated with bioinert materials. Additional functional ligands are then added to help with tumor recognition and targeted drug delivery (Lin and Zhang, 2023). High-resolution imaging of deep tissues is made possible by magnetic cobalt ferrite spinel (MCFS) nanoparticles, which are sophisticated MRI contrast agents that can enhance contrast in both T1-weighted positive and T2-weighted negative ways (Mikhaylov et al., 2022). MCFS nanoparticles have shown their dual utility in imaging and cancer treatment by being employed as nanocarriers for targeted drug delivery (Mikhaylov et al., 2022). Sophisticated molecular design and exact assembly techniques enable the nanoparticles to cross biological barriers and accumulate preferentially at tumor sites, thereby achieving this multifunctionality (Lin and Zhang, 2023).

The creation of multifunctional theranostic nano-agents by combining fluorescent dyes with discrete Pt (II) metallacycles is another creative example. High signal-to-background ratios and advantageous tumor distribution profiles are provided by these agents’ dual-modal imaging capabilities, which include photoacoustic imaging and second near-infrared (NIR-II) imaging (Sun et al., 2019). The nano-agents provide chemo-photothermal synergistic therapy guided by these imaging modalities, which leads to better antitumor performance and fewer side effects than stand-alone treatments (Sun et al., 2019)

Natural magnetic nanoparticles (nMNPs) derived from magnetotactic bacteria have been used as high-contrast photoacoustic probes and photothermal agents in triple-negative breast cancer, demonstrating how nanomaterials can be integrated with sophisticated imaging techniques to treat other cancer types (Nima et al., 2019). Low-energy laser pulses can be used to detect and photomechanically kill chemotherapy-resistant cancer cells thanks to these bioinspired hybrids, gold nanorods, and folic acid (Nima et al., 2019). These nanoparticles enable real-time tracking of circulating tumor cells in the bloodstream by facilitating super-resolution photoacoustic flow cytometry (Nima et al., 2019). These features demonstrate the adaptability of multifunctional nanomaterials in tackling the difficulties associated with cancer diagnosis and treatment, especially when traditional therapies prove ineffective.

Advances in ligand chemistry, which are essential for converting theoretical designs into functional nanosystems, further support the development of theranostic nanoparticles (Lin and Zhang, 2023). To ensure compatibility and synergistic functionality, capping ligands, drug-loading ligands, and targeting ligands are arranged hierarchically. Targeting ligands improve tumor specificity, and biodegradable moieties added to drug-loading ligands allow for intelligent drug release (Lin and Zhang, 2023). Personalized cancer medicine is made possible by this modular design approach, which enables nanoparticles to be tailored to the unique needs of various cancer types and treatment plans (Lin and Zhang, 2023). A paradigm shift in the treatment of cancer is represented by multifunctional nanomaterials that combine therapeutic and diagnostic properties. These nanoparticles provide previously unheard-of levels of safety, efficacy, and precision in the diagnosis and treatment of conditions like melanoma by fusing cutting-edge imaging methods with targeted drug delivery. Such theranostic platforms have enormous potential to improve patient outcomes and advance the field of nanomedicine if they are developed further, aided by advancements in ligand chemistry and materials science. To fully realize the potential of these multifunctional nanomaterials in personalized cancer therapy, future research should concentrate on improving their biocompatibility, scalability, and clinical translation.

## 5 Challenges in preclinical research and translation

Nanomaterial-based synergistic therapies have demonstrated significant potential in experimental studies; however, translating these therapies from the laboratory to the clinic remains fraught with challenges. This section will discuss the difficulties in clinical translation, current progress, and future prospects (Hassan et al., 2017).

Although the therapeutic efficacy of nanomaterials *in vivo* is promising, their long-term safety must be thoroughly evaluated. Some nanoparticles, particularly certain polymeric or inorganic materials, may exhibit poor physical stability or accumulation in the body, potentially leading to toxicity (Duan et al., 2018; Wu et al., 2022; Zeng H. et al., 2022). Reports have indicated that certain polymeric nanoparticles display higher toxic side effects in animal models, such as triggering inflammation or organ damage, which has impeded their clinical advancement (Zeng H. et al., 2022). Upon entering the bloodstream, nanoparticles can adsorb plasma proteins to form a protein corona, altering their distribution and clearance pathways and potentially activating the complement or coagulation systems (Shreffler et al., 2019). The immune system’s recognition and clearance of nanomaterials may also be stronger than anticipated, thereby reducing effective drug delivery and increasing the risk of immune-related adverse reactions (Shreffler et al., 2019; Czarnecka J. et al., 2020). Therefore, it is essential to optimize the material composition and surface properties of nanocarriers in preclinical studies to balance therapeutic efficacy with biocompatibility. This includes using more biodegradable and less toxic materials, as well as stable surface modifications, to ensure the safety of nanodrugs in humans.

The success of nanotherapies in animal models does not necessarily translate to similar efficacy in humans, and this cross-species translatability is one of the major challenges. On the one hand, the EPR effect in human tumors is highly variable, and not all patient tumors exhibit sufficient vascular permeability to allow efficient entry of nanodrugs (Barani et al., 2021; Moni et al., 2025). Some human melanomas have poor blood supply or dense stroma, which may result in lower delivery efficiency of nanodrugs compared to what is observed in mouse models (Moni et al., 2025). On the other hand, differences in the immune system and anatomical size between mouse models and humans may lead to new issues regarding the distribution, clearance, and immune interactions of nanodrugs in the human body. For example, some nanotherapeutic formulations that perform well in mice have been terminated in Phase II clinical trials due to insufficient efficacy or safety concerns (Corbo et al., 2016; Shreffler et al., 2019). Statistics show that the vast majority of nanodrugs that have entered clinical trials have yet to pass Phase II validation, which is closely related to the aforementioned translational difficulties (Shreffler et al., 2019). Therefore, enhancing the predictive power of animal studies, developing models that more closely resemble human conditions, and conducting more detailed pharmacokinetic and biodistribution studies are crucial for bridging the gap between experimental research and clinical application (Schäfer et al., 2022). Melanoma patients exhibit significant heterogeneity in the molecular characteristics and microenvironments of their tumors, making the optimization of nanosynergistic therapies for individual patients a complex challenge. Personalized treatment demands comprehensive molecular diagnostics of the patient’s tumor, followed by the tailoring of a bespoke therapeutic regimen. The inherent customizability of nanodrugs must align with this requirement. Tumors from different patients may necessitate distinct targeting ligands or drug combinations, imposing higher demands on the modular design and production of nanotherapeutics (Moni et al., 2025). However, in practice, this involves small-scale, customized manufacturing of nanodrugs, for which the processes, quality control, and regulatory frameworks are still underdeveloped.

## 6 Future perspectives

Despite the aforementioned challenges, several nanomaterials have already entered clinical trials for the treatment of melanoma. This study aims to leverage the synergy between nanochemotherapy and anti-angiogenic/immunotherapy to assess whether it can improve the response rate and survival of patients with advanced disease. However, to date, very few nanodrugs for melanoma have been truly approved by regulatory authorities. Most projects are still in Phase I/II trials, and more data are needed to demonstrate their superiority over standard treatments in terms of efficacy and safety.

With the integration of nanotechnology with other cutting-edge therapies (such as gene editing and CAR-T cell therapy), more innovative clinical strategies are expected to emerge in the future (Li et al., 2019; Lakshmanan et al., 2021; Xi et al., 2022). It can be anticipated that future clinical evaluations of nanosynergistic therapies will place greater emphasis on optimizing combination therapies, such as selecting the optimal nanodose and timing of immunotherapy, and identifying biomarkers that can predict response (Dai et al., 2022; Nguyen et al., 2022; Overchuk et al., 2023). This will help determine which patients are most likely to benefit from specific nanosynergistic regimens.

In order to improve antitumor effects in a murine model of melanoma, a study investigates the creation of a novel immunogenic compound, SZU-119, which combines a TLR7 agonist (SZU-101) with the small-molecule BRD4 inhibitor JQ-1 (Wang X. et al., 2021). *In vitro*, the compound, which was created by chemical conjugation, showed promise in activating TLR7 signaling and suppressing PD-L1 expression. *In vivo* tests showed that SZU-119 increased the infiltration of CD8^+^ cytotoxic T cells into tumors, prolonged mouse survival, and significantly suppressed tumor growth at both injected and uninjected sites. By simultaneously targeting innate and adaptive immune responses, this study demonstrates SZU-119’s potential as a promising agent for melanoma immunotherapy and provides fresh information for the creation of anti-melanoma medications (Wang X. et al., 2021).

A new nanocomposite (NC) for multimodal cancer treatment that combines immune checkpoint blockade therapy (ICBT), chemotherapy, and PTT to improve antitumor efficacy and create long-term immune memory (Mei et al., 2022). The NC is made up of anti-PD-L1 antibodies (aPD-L1) for targeted immune therapy, polymetformin (PolyMet) as a chemotherapeutic agent, and black phosphorus nanosheets (BPN) as a photothermal agent. By using BPN’s photothermal effect to create localized hyperthermia, the combination improves drug release and increases PD-L1 expression in tumor cells, allowing aPD-L1 to target them precisely. Synergistic antitumor effects result from this positive feedback mechanism, which increases the NC’s accumulation in tumor sites (Mei et al., 2022).

Another study uses the small molecule inhibitor JQ1 to investigate the therapeutic potential of BET bromodomain inhibition in castration-resistant prostate cancer (CRPC) (Asangani et al., 2014). It demonstrates how sensitive CRPC cells with active androgen receptor (AR) signaling are to JQ1, which interferes with BRD4-AR interaction, preventing AR-mediated gene transcription and slowing tumor growth. JQ1 blocks AR target gene expression and BRD4 localization more successfully than direct AR antagonists because it acts downstream of AR. In xenograft models, JQ1 treatment outperforms MDV3100 in terms of tumor weight and volume reduction. This study highlights BET bromodomain inhibitors’ potential as a novel therapeutic approach for CRPC and raises the possibility that similar strategies could be helpful in other cancers, such as melanoma, where transcriptional dysregulation is a major driver (Asangani et al., 2014).

The new uses of violet phosphorus (VP) nanomaterials in biomedicine are highlighted in a review paper (Mei et al., 2025). In comparison to black phosphorus, VP, including bulk VP, VP nanosheets (VPNs), and VP quantum dots (VPQDs), offers special qualities like a tunable bandgap, good biodegradability, and increased stability. Because of these properties, VP nanomaterials hold promise for biosensing, antibacterial therapy, and anticancer therapy (Mei et al., 2025). Notwithstanding these developments, there are still issues with clinical translation, thorough property exploration, and scalable production. In order to fully utilize VP nanomaterials in biomedical applications, including the treatment of melanoma, these problems must be resolved.

Reprogramming the TME with biomineralized bacterial outer membrane vesicles (OMVs) to improve cancer immunotherapy. Gram-negative bacteria are the source of OMVs, which have immunostimulatory components but have drawbacks like quick clearance and toxicity when administered intravenously (Qing et al., 2020). In order to solve these problems, scientists created OMV@CaPs by encasing OMVs in calcium phosphate (CaP) shells (Qing et al., 2020). These CaP shells dissolved in the acidic TME, neutralizing the tumor pH and encouraging M2-to-M1 macrophage polarization, in addition to protecting OMVs from immune clearance. In mouse models, the OMV@CaPs dramatically reduced tumor growth and increased survival rates. The study also showed how OMV@CaPs could be functionalized with photosensitizers and targeting ligands to improve tumor targeting and photothermal therapy. This adaptable platform presents a viable approach to overcoming immunosuppressive TMEs and enhancing the results of cancer treatment.

Future nanotherapies will become increasingly intelligent and autonomous, capable of self-regulation in response to changes in the tumor microenvironment. Some researchers have proposed leveraging artificial intelligence (AI) to design nanoparticles, incorporating vast amounts of biological data into models to optimize the formulation and dosing regimens of nanodrugs, thereby achieving AI-driven nanoprecision medicine. With the advancement of tumor molecular diagnostics, the mutational profiles and immune landscapes of individual melanoma patients will become increasingly clear. Future nanotherapies can be integrated with such precise diagnostics to form truly patient-centered individualized treatment plans. The future of nanomedicine will also benefit from the convergence of materials science, bioengineering, and computer science. Novel nanomaterials, such as DNA origami nanostructures, self-assembling peptides, and hydrogel microparticles, will continue to emerge, providing additional tools for synergistic therapy. Nanorobots and actively motile nanocarriers are also under exploration, with the potential to autonomously navigate to tumors within the body and enhance delivery efficiency. Interdisciplinary collaboration will help address some of the current bottlenecks in nanotherapy, such as achieving more sensitive *in vivo* monitoring with new materials like quantum dots and safer carrier degradation with biodegradable materials.

Nanomaterial-driven synergistic therapy is paving new directions for the comprehensive treatment of melanoma. Mechanistically, it overcomes the limitations of monotherapy by delivering multi-level attacks at the molecular, cellular, and organ levels. In terms of efficacy, preclinical studies have demonstrated significant potential in improving therapeutic outcomes, overcoming drug resistance, and reducing recurrence. Of course, clinical translation still faces many challenges, including addressing safety, manufacturing, and regulatory issues. However, with scientific progress and multidisciplinary collaboration, these barriers are expected to be gradually overcome.
